# Primary anterior cruciate ligament repair with suture tape augmentation: indications, failure predictors, clinical outcomes, and evidence gaps

**DOI:** 10.3389/fspor.2026.1893928

**Published:** 2026-09-16

**Authors:** Ibrahim Alabid, Hesham Amin Hamdy, Mohamedanas Mohamedfaruk Patni, Ali Jad Yousef, Biji Thomas George, Aiswarya Menon, Khaled Walid Elayyan

**Affiliations:** 1 RAK College of Medical Sciences, Ras Al Khaimah Medical and Health Sciences University (RAKMHSU), Ras Al Khaimah, United Arab Emirates; 2 Department of Surgery, RAK College of Medical Sciences, RAK Medical and Health Sciences University, Ras Al Khaimah, United Arab Emirates; 3 Department of Community Medicine, RAK College of Medical Sciences, RAKMHSU, Ras Al Khaimah, United Arab Emirates

**Keywords:** anterior cruciate ligament, bridge-enhanced ACL restoration, failure predictors, internal bracing, primary ACL repair, proximal ACL tear, return to sport, suture tape augmentation

## Abstract

**Background:**

Primary anterior cruciate ligament (ACL) repair has re-emerged as a tissue-preserving option for selected proximal tears. Suture tape augmentation may protect the repaired ligament during early healing, but its clinical role remains uncertain because outcomes vary across patient groups and surgical techniques.

**Methods:**

A narrative review of PubMed, Scopus, Google Scholar, and reference lists was conducted to identify relevant clinical, imaging, arthroscopic, and biomechanical studies published mainly from 2015 to May 2026.

**Results:**

The strongest indications for repair are acute Sherman type I and selected type II proximal tears with an adequate, reducible remnant, good tissue quality, and preserved synovial coverage. Biomechanical evidence supports improved time-zero stability with suture tape augmentation. Clinical studies report satisfactory short- to midterm outcomes in selected adults, whereas younger patients, high-demand athletes, military populations, delayed presentations, and less favorable tear characteristics have higher failure rates. Bridge-enhanced ACL restoration represents a distinct scaffold-assisted preservation strategy and was considered separately.

**Conclusions:**

Primary ACL repair with suture tape augmentation is a selective preservation strategy rather than a general substitute for reconstruction. Larger comparative studies using standardized indications, techniques, rehabilitation protocols, and failure definitions are needed to establish its long-term role.

## Graphical Abstract

**Figure:**
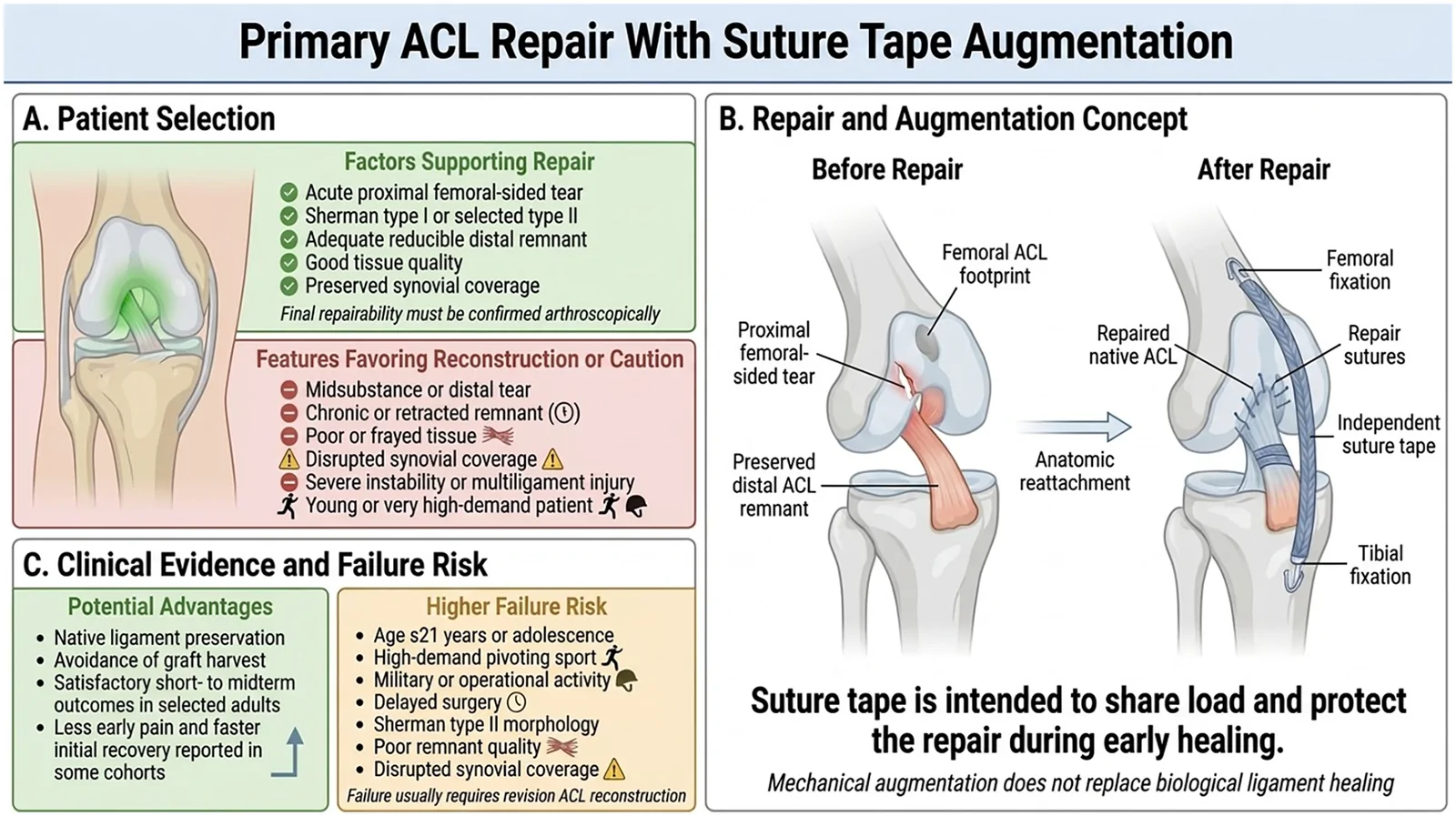
Overview of patient selection, repair technique, potential advantages, and failure-risk factors for primary ACL repair with suture tape augmentation. ACL, anterior cruciate ligament; ACLR, anterior cruciate ligament reconstruction. Created in BioRender. Alabid, I. (2026) https://BioRender.com/u0ldcqi.

## 1 Introduction

Anterior cruciate ligament (ACL) rupture is a common and clinically important knee injury that compromises rotational and anteroposterior stability, limits participation in sports and demanding activities, and may increase the risk of secondary meniscal and chondral injury. ACL reconstruction remains the standard operative treatment for patients with symptomatic instability because historical primary repair produced inconsistent midterm stability and durability. However, reconstruction replaces rather than preserves the native ligament and may be associated with graft-harvest morbidity, altered proprioception, residual symptoms, and incomplete return to preinjury activity (1–3).

The unfavorable outcomes of early ACL repair were influenced not only by the biological limitations of ligament healing but also by the broad application of repair to unsuitable tear patterns. Historical techniques frequently included midsubstance ruptures, retracted or poor-quality remnants, open surgery, nonanatomic fixation, and prolonged postoperative immobilization. Contemporary repair differs substantially from these earlier approaches. Modern arthroscopic techniques use preoperative magnetic resonance imaging and direct intraoperative assessment to identify tears with characteristics more suitable for preservation, particularly proximal femoral-sided ruptures with adequate remnant length, good tissue quality, preserved synovial coverage, and the ability to restore the ligament to its anatomic femoral footprint (1–5).

Renewed interest in primary ACL repair has also been driven by advances in fixation and augmentation. Suture-anchor techniques permit direct reattachment of the ACL remnant, while suture tape augmentation provides an independent secondary construct intended to share load and protect the repair during the early healing period. These developments may reduce early elongation and gap formation, which are important mechanical concerns after primary repair. Nevertheless, augmentation should be viewed as protection for a biologically repairable ligament rather than a method for extending repair to unsuitable tear patterns (6–11).

Current clinical evidence suggests that satisfactory outcomes may be achieved in appropriately selected patients, but results remain variable across techniques and populations. Younger patients, adolescents, and individuals exposed to high-demand pivoting or occupational activity appear to have greater failure risk, emphasizing that technical repairability does not necessarily guarantee clinical durability (6–8). Moreover, differences in tear classification, patient selection, surgical technique, rehabilitation, follow-up duration, and definitions of failure make direct comparison between studies difficult.

This narrative review evaluates the indications and contraindications for primary ACL repair, the biological and biomechanical rationale for suture tape augmentation, clinical and comparative outcomes, failure predictors, revision considerations, and major evidence gaps. Particular attention is given to distinguishing static suture tape augmentation from other ACL-preservation techniques and to defining the clinical circumstances in which repair may be considered instead of reconstruction.

## 2 Literature search strategy

A structured literature search was performed using PubMed, Scopus, Google Scholar, and manual reference screening. The search focused mainly on studies published from January 2015 to May 2026. The 2015 starting point was chosen to focus the review on the contemporary era of arthroscopic ACL preservation and modern augmentation techniques. Earlier landmark studies were retained when necessary to explain the historical transition from primary repair to reconstruction, foundational surgical concepts, and the rationale for modern ACL preservation. The search strategy combined free-text terms and Boolean operators, including: “primary ACL repair,” “anterior cruciate ligament repair,” “ACL preservation,” “internal brace,” “suture tape augmentation,” “suture ligament augmentation,” “proximal ACL tear,” “Sherman type,” “dynamic intraligamentary stabilization,” “ACL repair failure,” “ACL repair vs. reconstruction,” “return to sport,” “MRI healing,” “second-look arthroscopy,” and “biomechanical ACL repair.” Original clinical studies were prioritized, including randomized trials, comparative cohort studies, prospective and retrospective cohorts, case series, imaging studies, second-look arthroscopic studies, and biomechanical laboratory studies. Studies were included when they directly addressed primary ACL repair, internal bracing or suture tape augmentation, dynamic intraligamentary stabilization, tear repairability, failure predictors, return to sport, postoperative healing, or revision concerns. Pure technique notes were not prioritized unless they clarified the technical evolution of repair. Non-ACL ligament studies and studies unrelated to repair or preservation were excluded. Because of heterogeneity in repair techniques, patient-selection criteria, follow-up duration, outcome measures, and failure definitions, no formal meta-analysis was performed. Because this was a narrative review incorporating heterogeneous study designs, no formal study-level risk-of-bias tool was applied. A single instrument such as ROBINS-I would not be applicable across all included designs, which comprised randomized trials, observational cohorts, case series, imaging and arthroscopic studies, and biomechanical experiments. Methodological limitations of individual studies were instead considered qualitatively during evidence synthesis. Findings were synthesized narratively and organized into clinically relevant domains: evolution and rationale, indications, clinical outcomes, failure predictors, revision concerns, clinical positioning, and future evidence gaps. BEAR was screened as a related ACL-preservation strategy but was analyzed separately from the principal synthesis of static suture tape augmentation and DIS to avoid combining clinically and technically distinct preservation approaches within the same evidence framework. The complete reference list was audited against the original publisher records and PubMed-indexed metadata where available. For each reference, the authors verified the author order, article title, journal, publication year, volume, issue, pagination or article number, and DOI. The study design, population, intervention, follow-up duration, and numerical outcomes cited in the manuscript were subsequently cross-checked against the corresponding original article. Related publications arising from the same research programme or potentially overlapping cohorts were identified and were not treated as independent evidence when interpreting the literature. Bibliographic metadata obtained from automated reference software were not accepted without comparison with the version of record. Because this work was designed as a narrative review rather than a systematic review, study identification and selection were not conducted as a protocolized PRISMA screening process; therefore, a PRISMA flow diagram was not generated.

## 3 Evolution and rationale of modern primary ACL repair

Primary anterior cruciate ligament (ACL) repair is not a new concept, but its modern form differs substantially from the historical open repairs that were largely abandoned in favor of reconstruction. Earlier repair techniques were applied broadly to different tear patterns, including midsubstance and poor-quality ruptures, and were commonly performed through open arthrotomy with prolonged postoperative immobilization. These factors likely contributed to stiffness, persistent instability, and deterioration of outcomes over time. Modern arthroscopic repair, by contrast, is based on a selective preservation concept: the torn native ACL is reapproximated to its femoral footprint only when the remnant is proximal, reducible, and mechanically suitable for suture fixation (1–3). Contemporary repair differs from historical repair through arthroscopic visualization, imaging-based selection, anatomic fixation, and early controlled rehabilitation (1–3).

The central principle of contemporary primary ACL repair is native ligament preservation. Unlike ACL reconstruction, which replaces the torn ligament with an autograft or allograft, repair attempts to preserve the patient's own ACL fibers, restore continuity to the femoral footprint, and potentially maintain native proprioceptive and biological functions. This rationale is particularly relevant for proximal avulsion-type tears, in which the distal remnant remains long enough to be advanced and fixed back to the femoral origin. Modern direct repair is primarily applied when the distal ACL remnant can be reduced securely to the femoral footprint and can tolerate suture fixation (1, 2). In skeletally immature patients, the same preservation logic has additional theoretical value because repair may avoid physeal tunnel-related risks associated with some reconstructive techniques, although the evidence in children remains limited and highly selected (12).

Modern repair has evolved into several related but distinct technical strategies. Suture-anchor repair uses sutures passed through the ACL remnant and fixed to the femoral footprint with anchors. Static suture tape augmentation, also described generically as internal bracing, adds an independent high-strength tape construct as a secondary stabilizer intended to share load and protect the repair during early healing. In this review, suture tape augmentation and internal bracing are used as generic terms for an independent high-strength tape construct that supplements primary ACL repair. InternalBrace™ is a proprietary Arthrex term and is used only when referring specifically to the branded Arthrex technique or when reproducing the title of a published article. Unless otherwise stated, discussion of internal bracing in this review refers to the generic concept of suture tape augmentation rather than a specific commercial implant. Dynamic intraligamentary stabilization (DIS) uses a different implant concept, in which a dynamic spring-screw mechanism maintains tension during healing. These approaches all aim to preserve the ACL, but they should not be interpreted as identical procedures because their fixation methods, biomechanics, rehabilitation implications, and revision concerns differ (6, 7, 13–16). This distinction is important when interpreting the literature because pooled discussion of “ACL repair” can obscure clinically meaningful differences between static internal bracing, suture-anchor repair, and DIS. For scope and interpretability, BEAR is treated as a related but separate ACL-preservation strategy and is not combined directly with the static suture tape augmentation or DIS evidence summarized in the principal clinical tables. The generic procedural sequence of primary ACL repair with independent suture tape augmentation is illustrated in Figure 1.

**Figure 1:**
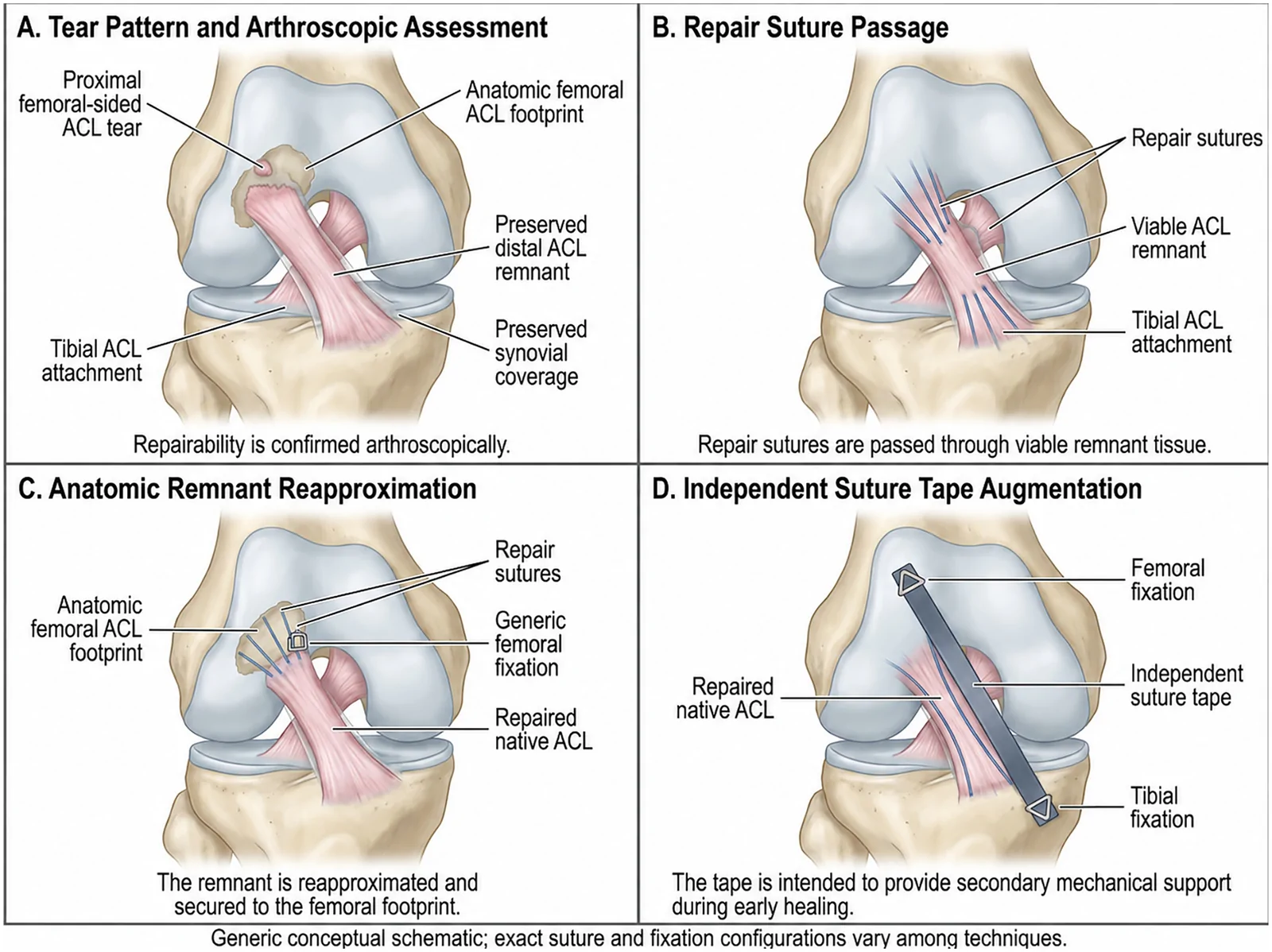
Generic procedural sequence of primary ACL repair with independent suture tape augmentation. **(A)** Arthroscopic assessment of a proximal femoral-sided ACL tear with a preserved distal remnant and synovial coverage. **(B)** Passage of repair sutures through viable remnant tissue. **(C)** Anatomic reapproximation and fixation of the ACL remnant to the femoral footprint. **(D)** Addition of an independent femoral-to-tibial suture tape construct alongside the repaired native ACL. Exact suture and fixation configurations vary among techniques. ACL, anterior cruciate ligament. Created in BioRender. Alabid, I. (2026) https://BioRender.com/0h8ay0l.

The biological rationale for repair depends strongly on tear morphology and tissue quality. MRI-based classification studies have shown that proximal tears represent a meaningful subset of ACL ruptures. In a prevalence study of 353 adult acute ACL tears, type I proximal avulsion tears represented 16%, type II proximal tears 27%, and type III midsubstance tears 52%; type I tears were also more common in patients older than 35 years than in younger patients (4). These findings suggest that repairable patterns are not rare, but they also show that most ACL tears remain midsubstance injuries and therefore are less suitable for direct femoral reattachment (4).

Tissue quality is equally important. Preoperative MRI can help estimate repairability, but the final decision remains intraoperative because the surgeon must confirm that the remnant has sufficient length, can be advanced to the femoral wall, and can withstand suture passage. In an MRI eligibility study, repair patients more often had type I tears and good tissue quality than reconstruction patients; 90% of MRI type I tears and 88% of type II tears with good tissue quality were repaired, whereas type II tears with poor tissue quality and most type III tears required reconstruction (17). A later remnant-length study further refined this concept by showing that tear location could be quantified reliably on MRI and that a proximal-quarter tear had a high positive predictive value for repairability (18).

Preserved synovial coverage may support ACL healing by maintaining a biologically favorable envelope around the torn ligament. Conversely, disruption of the synovial sheath may impair healing and contribute to residual instability or failure (19).

The biomechanical rationale for internal bracing is based on the concern that primary repair alone may be vulnerable to early elongation before biological healing is complete. Gap formation at the repair site may reduce femoral contact, impair scar formation, and contribute to residual laxity. In a controlled laboratory study, Bachmaier et al. showed that different fixation constructs varied in time-zero gap formation, residual load-bearing capability, and ultimate strength; adjustable cortical button fixation with preconditioning reduced gap formation and had the highest failure load among the tested nonaugmented techniques (9). In the companion study, internal bracing improved stabilization, reduced peak loads on the ACL repair construct, and restricted gap formation to below 3 mm at loads up to 350 N (10).

Human cadaveric biomechanical data also support the mechanical value of suture tape augmentation. He et al. found that ACL suture repair alone did not restore intact knee anterior tibial translation or *in situ* ACL forces, whereas adding suture tape reduced laxity and brought knee kinematics closer to the intact ACL state; fixation at 20° of knee flexion performed better than fixation in full extension under pivot-shift and internal-rotation loading (11). Similarly, Muench et al. compared cortical or anchor fixation with suture tape augmentation against bone–patellar tendon–bone ACL reconstruction in a cadaveric model, adding further biomechanical context to repair-augmentation strategies (20). Together, these studies suggest that internal bracing should be viewed as mechanical protection for a biologically selected repair, not as a method to make all ACL tear types repairable.

## 4 Indications and patient selection

Patient selection is the defining determinant of success in primary ACL repair. The strongest indication is an acute proximal femoral-sided tear, particularly Sherman type I proximal avulsion and selected type II proximal tears, with a remnant that can be reduced to the femoral footprint and has sufficient tissue quality to hold sutures. This approach is supported by imaging and candidate-selection studies showing that proximal tear location, remnant length, and tissue quality predict repairability (4, 5, 17, 18). In contrast, midsubstance tears, distal tears, chronic tears, retracted remnants, and poor-quality tissue are generally less suitable for primary repair and should usually be treated with reconstruction.

Preoperative MRI is central to identifying potential candidates. The modified Sherman classification divides ACL tears according to remnant length: type I proximal avulsion tears have more than 90% distal remnant length, type II proximal tears have 75%–90%, type III midsubstance tears have 25%–75%, type IV distal tears have 10%–25%, and type V distal avulsion tears have less than 10% (4). In the MRI prevalence study, 43% of ACL tears were in the proximal quarter, but the majority were still midsubstance tears, which reinforces that repair should be considered selectively rather than as a universal strategy (4).

MRI can also help estimate whether a tear is technically repairable. In the preoperative MRI eligibility study, repair was much more likely when the tear was type I or type II with good tissue quality. All patients with type I tears and good tissue quality were repaired; among type II tears, 88% with good tissue quality were repairable, compared with 23% with fair tissue quality and none with poor tissue quality (17). These findings show that tear location alone is insufficient: repairability depends on both location and remnant quality. The remnant-length study further demonstrated that all patients with tear location ≥80% on MRI were eligible for repair, whereas those with tear location <60% required reconstruction; a tear location ≥75% was highly predictive of repairability (18).

Despite the value of MRI, the final indication is intraoperative. The surgeon must confirm that the remnant is reducible, not excessively frayed or retracted, and strong enough to tolerate suture passage and tensioning. Final repairability should be confirmed arthroscopically by assessing remnant length, tissue integrity, reducibility, and the ability of the tissue to tolerate suture passage and tensioning (1, 17). Therefore, a practical repair strategy should involve preoperative planning for repair but readiness to convert to reconstruction if remnant length, tissue quality, reducibility, or synovial integrity is inadequate.

Synovial coverage should be incorporated into candidate selection. Although preoperative imaging may suggest preservation of the synovial envelope, final assessment remains arthroscopic. Primary repair is more appropriate when the synovial sheath is intact or substantially preserved, whereas marked disruption or multilaceration should favor reconstruction. Second-look arthroscopy findings also support synovial coverage as a practical marker of healing quality (19, 21).

Timing from injury to surgery is another important consideration. Acute and subacute repairs are generally favored because tissue quality, remnant mobility, and healing potential may decline with time. In the candidate-selection case–control study, surgery within 28 days was independently associated with the possibility of primary repair, along with age greater than 35 years and body mass index below 26 (5). Ferretti et al. evaluated acute ACL repairs performed within 14 days and reported early clinical and MRI healing findings in patients with proximal tears and good tissue quality (22). Therefore, repair is best considered early, before remnant scarring, retraction, or tissue degeneration reduces repairability.

Age and anticipated activity demand should be incorporated into candidate selection. Repair may be feasible in some skeletally immature patients with proximal avulsion tears, but adolescents, patients aged 21 years or younger, and individuals exposed to high-demand pivoting, elite sporting, or military activity appear to have less reliable durability (8, 12, 23, 24). Activity level should therefore be considered together with tear morphology, tissue quality, and timing rather than as an isolated criterion (24, 25).

Concomitant injuries may influence both the indication and technique. Meniscal tears are common and may be repaired or treated during the same procedure, but lateral meniscus injury was associated with a lower likelihood of primary repair in the candidate-selection study (5). Combined anterolateral pathology may also be relevant. Hopper et al. reported outcomes after combined ACL repair and anterolateral ligament suture tape augmentation in patients with indications such as Segond fracture, grade 3 pivot shift, or high sporting activity, suggesting that rotational instability may require additional consideration rather than isolated ACL repair alone (26). Meniscal repair with concomitant suture-augmented ACL repair has also been compared with meniscal repair with ACL reconstruction in recent evidence, but this area remains early and should be interpreted cautiously (27).

Candidate selection should therefore integrate tear location, remnant length and reducibility, tissue quality, synovial coverage, injury chronicity, associated pathology, patient age, and anticipated activity demand. A practical framework summarizing the principal indications, contraindications, intraoperative decision points, and recognized failure predictors is presented in Table 1.

**Table 1:** Clinical decision framework for primary ACL repair with suture tape augmentation.

Domain	Findings supporting repair	Findings favoring reconstruction or caution	Failure implication
Tear location	Proximal femoral-sided Sherman type I; selected type II	Midsubstance, distal, or multilacerated tear	Nonproximal morphology reduces repairability and may increase failure
Remnant length	Adequate distal remnant that reaches the femoral footprint	Short, retracted, or immobile remnant	Inability to restore footprint contact is a reason to abandon repair
Tissue quality	Strong tissue capable of holding sutures	Frayed, attenuated, or degenerative tissue	Poor tissue increases elongation and biological failure
Reducibility	Remnant can be reapproximated without excessive tension	Remnant cannot be reduced anatomically	Nonanatomic or tensioned repair is unsuitable
Synovial coverage	Intact or preserved synovial sheath	Disrupted or multilacerated synovial envelope	Synovial disruption is associated with higher failure
Timing	Acute or early subacute injury	Chronic tear with scarring or retraction	Delay may reduce mobility and tissue quality
Age	Skeletally mature adult	Adolescent or patient aged ≤21 years	Young age is consistently associated with higher failure
Activity demand	Moderate athletic or occupational demand	Elite pivoting sport, military, or operational demand	Repetitive high-load exposure increases rerupture risk
Associated injuries	Treatable meniscal pathology without severe global instability	Multiligament injury, severe rotational instability, or major associated pathology	Additional instability may make isolated repair inadequate
Intraoperative assessment	Good tissue, adequate length, stable anatomic reattachment	Uncertain fixation, poor tissue, excessive laxity	Convert to ACL reconstruction when repairability is uncertain

Final repairability should be confirmed arthroscopically. Preoperative MRI may identify a potentially repairable proximal tear, but ACL reconstruction should be performed when remnant length, tissue quality, reducibility, synovial integrity, or fixation security is inadequate. ACL, anterior cruciate ligament; MRI, magnetic resonance imaging.

## 5 Clinical evidence

The clinical evidence for primary ACL repair has progressed from small proof-of-concept case series to larger cohorts, comparative studies, randomized trials, imaging studies, and recent midterm follow-up reports. However, interpretation requires caution because studies differ in technique, tear selection, patient age, activity level, follow-up, and definition of failure. Table 2 summarizes the key clinical studies representing repairability, suture tape augmentation outcomes, comparative evidence, failure predictors, and high-risk populations. BEAR is not included in Tables 2, 3 because it was intentionally treated as a related but distinct ACL-preservation strategy; its evidence is summarized separately in Section 8.1. Early suture-anchor studies established the clinical feasibility of modern selective ACL repair. In the original DiFelice cohort, 10 of 11 patients had satisfactory clinical outcomes at a mean follow-up of 3.5 years, and these outcomes were largely maintained among the 10 patients available at a mean 6-year follow-up (1, 3). Detailed study characteristics and outcomes are presented in Table 2.

**Table 2:** Characteristics of key clinical studies evaluating primary ACL repair with or without suture tape augmentation.

Study	Design/level	Population/sample	Technique/comparison	Follow-up	Main outcomes/key findings	Why this study is important
DiFelice et al. (1)	Retrospective case series; Level IV	11 patients with proximal avulsion ACL tears and excellent tissue quality	Arthroscopic suture-anchor primary ACL repair	Minimum 2 years; mean 3.5 years	10/11 had good outcomes. Mean Lysholm 93.2, modified Cincinnati 91.5, SANE 91.5, subjective IKDC 86.4. Objective IKDC A in 9/11. One clinical failure. No postoperative complications or additional surgery reported.	Foundational proof-of-concept study demonstrating successful repair in proximal avulsion tears with excellent tissue quality.
van der List et al. (4)	MRI prevalence study; Level IV	353 adult acute ACL tears on MRI	MRI classification of ACL tear location	Imaging study	43% of tears were in the proximal quarter: 16% type I and 27% type II. Type III midsubstance tears were most common at 52%. Type I tears were more common in older patients.	Supports the rationale that repairable proximal tear patterns exist in a meaningful subset of ACL injuries.
van der List et al. (5)	Retrospective case-control study	361 ACL surgery patients; 158 repair, 203 reconstruction	Primary repair if proximal tear with good tissue quality; otherwise, ACL reconstruction	Not primarily an outcomes study	Primary repair was possible in 44%. Independent predictors of repairability were age >35 years, surgery within 28 days, BMI <26, while lateral meniscus injury reduced repair likelihood.	Best paper for candidate selection and practical preoperative predictors of repairability.
Ateschrang et al. (19)	Cohort study; Level IV	124 patients with proximal ACL tears treated with DIS	DIS; grouped by synovial sheath integrity	Mean 2.3 ± 0.8 years	Overall failure 17.7%. Failure was 4% in one-part tears with intact synovial coverage versus 26.9% in two-part tears and 27.3% in multilacerated tears. Synovial disruption was independently associated with failure.	Strongest study supporting synovial sheath integrity as a biological selection factor.
Hoogeslag et al. (28)	Randomized controlled trial; Level I	48 patients with acute ACL rupture	DIS vs single-bundle all-inside ACL reconstruction	2 years	DIS was noninferior to reconstruction for IKDC subjective score. Median IKDC was 95.4 in repair vs 94.3 in reconstruction. Rerupture occurred in 2 repair patients and 4 reconstruction patients, but repeat surgery for other reasons was higher in repair.	One of the highest-quality comparative trials supporting short-term noninferiority, but also showing procedure-related reoperation concerns.
Heusdens et al. (29)	Prospective interventional case series; Level IV	35 patients with repairable ACL rupture, 4–12 weeks old	Suture tape augmentation ACL repair	2 years	Four reruptures, giving 11.4% rerupture rate. Three additional reinterventions. MRI showed grade 1 healing in 45.2%, grade 2 in 35.5%, and grade 3 in 19.4%. Grade 3 MRI healing and preoperative Tegner ≥7 were associated with higher rerupture risk.	Important because it combines clinical outcomes, MRI healing, and rerupture predictors after suture tape repair.
Hopper et al. (30)	Prospective case series; Level IV	37 consecutive patients with acute proximal ACL rupture; 34 available at final analysis	Primary ACL repair with suture tape augmentation	Minimum 5 years	Mean KOOS improved from 48.7 to 88.5. Six patients reruptured, giving 17.6% rerupture. Rerupture patients were younger and had higher initial Marx activity scores.	Key midterm suture tape study; shows good PROMs but meaningful rerupture risk over time.
Cruz et al. (24)	Case series; Level II in article	46 active-duty military patients	ACL repair with suture tape augmentation	Minimum 2 years	Failure occurred in 12/46 patients, 26.1%. Higher failure was associated with high competition/operational level and tobacco use; failure group also had longer injury-to-surgery interval.	Best high-demand population study; essential for the “failure predictors” section.
Glasbrenner et al. (16)	Randomized controlled trial; Level I	85 randomized patients; 64 available at 5 years	DIS vs ACL reconstruction	5 years	At 5 years, recurrent instability occurred in 35% of DIS patients and 20% of reconstruction patients. Recurrent instability was associated with younger age and higher preinjury Tegner activity.	Best longer-term randomized evidence; shows similar PROMs/laxity among successful patients but important recurrent instability risk.
Al Kindi et al. (31)	Prospective single-cohort study	61 primary ACL repairs; 54 completed minimum 3-year follow-up	Primary ACL repair in selected patients ≤50 years with recent ACL injury	Minimum 3 years; average 49.6 months	Overall success 82.2%. Sherman type I tears had fewer failures than type II tears: 4.3% vs 31.8%. Mean Lysholm in successful repairs was 96. Adolescents had the highest failure rate at 50%.	Recent prospective evidence showing a lower failure rate in Sherman type I than type II tears.

The table highlights representative studies addressing repairability, patient selection, clinical outcomes, comparative evidence, failure predictors, and midterm durability. ACL, anterior cruciate ligament; ACLR, anterior cruciate ligament reconstruction; DIS, dynamic intraligamentary stabilization; IKDC, International Knee Documentation Committee; KOOS, Knee injury and Osteoarthritis Outcome Score; MRI, magnetic resonance imaging; PROMs, patient-reported outcome measures; RCT, randomized controlled trial; STA, suture tape augmentation.

**Table 3:** Additional midterm and comparative studies evaluating primary ACL repair with suture tape augmentation.

Study	Design and population	Technique/comparator	Follow-up	Main findings	Main limitations
Hopper et al. (30)	Prospective case series; 37 patients enrolled and 34 assessed	Primary ACL repair with suture tape augmentation	Minimum 5 years	Mean KOOS improved from 48.7 to 88.5; six reruptures occurred; patients with rerupture were younger and had higher initial activity scores	Uncontrolled study with a small selected cohort
Hopper et al. (32)	Retrospective comparative cohort; 134 repairs and 272 reconstructions	Primary repair with suture tape augmentation versus ACL reconstruction	Clinical follow-up	No statistically significant difference in overall secondary surgery; rerupture was numerically higher after repair	Nonrandomized treatment selection and unequal groups
Douoguih et al. (33)	Case series; 27 proximal avulsion or high-grade partial avulsion tears	Primary ACL repair with suture augmentation	Mean 2.8 years	Four recurrent ACL injuries required revision reconstruction	Small cohort without a reconstruction control group
Schneider et al. (34, 35)	Related clinical outcome and revision-risk cohorts	Primary ACL repair with suture augmentation	Mean 21 months to 4 years	Satisfactory patient-reported outcomes were reported; revision-free survival was approximately 90% at 4 years	Probable overlap between the reported cohorts
Szwedowski et al. (36)	Small retrospective comparative study	Primary repair with additional internal bracing versus ACL reconstruction	Short-term follow-up	Anterior tibial translation was lower after repair, while Lysholm scores did not differ significantly	Small sample, retrospective design, and possible selection bias
Müller et al. (37)	Matched comparative study	Arthrex InternalBrace™ repair versus ACL reconstruction and healthy controls	2 years	Comparable patient-reported, clinical, and functional outcomes; operative time was shorter after repair	Selected repairable tears and limited follow-up
Duong et al. (38)	Comparative clinical, MRI, and patient-reported outcome study	Primary ACL repair versus ACL reconstruction	Short-term follow-up	Selected repairs achieved comparable clinical, imaging, and patient-reported outcomes	Nonrandomized selection and matching limitations
Simard et al. (39)	Three-group comparative cohort	Proximal repair with suture tape augmentation, early reconstruction with augmentation, and standard reconstruction	2 years	Similar short-term clinical outcomes and side-to-side laxity across groups	Nonrandomized treatment allocation and selected populations

The studies summarized in this table differed in tear selection, augmentation technique, comparator group, follow-up duration, and definition of failure. Similar outcomes in selected repair cohorts should therefore not be interpreted as evidence that primary ACL repair is equivalent to reconstruction for all ACL injuries. ACL, anterior cruciate ligament; ACLR, anterior cruciate ligament reconstruction; IKDC, International Knee Documentation Committee; KOOS, Knee injury and Osteoarthritis Outcome Score; MRI, magnetic resonance imaging; PROMs, patient-reported outcome measures.

Pediatric and skeletally immature evidence remains limited. Bigoni et al. reported five Tanner stage 1–2 patients treated with arthroscopic suture-anchor reinsertion for proximal tears. At a mean follow-up of 43.4 months, no reinjury, leg-length discrepancy, or growth arrest was observed, and all patients returned to previous activity with a mean Lysholm score of 93.6 (12). Although this supports feasibility in very selected children, the sample is too small to establish safety across the broader adolescent population, especially given later evidence of high failure risk in adolescents treated with suture ligament augmentation (8).

Studies of primary ACL repair with suture tape augmentation provide the main evidence base for the technique addressed in this review. Jonkergouw et al. evaluated 56 consecutive arthroscopic primary repairs of proximal ACL tears, with the latter 27 patients receiving additional internal bracing. At a mean 3.2-year follow-up, 6 repairs failed, giving a failure rate of 10.7%; failure was numerically lower with internal bracing than without it, but the difference was not statistically significant (6). Heusdens et al. reported 42 patients treated with independent suture tape reinforcement, with significant improvements in KOOS, VAS pain, and VR-12 physical scores and 2 reruptures at 2-year follow-up (7). These studies demonstrated improvements in patient-reported outcomes, but also showed that internal bracing does not eliminate failure risk (6, 7).

Longer follow-up of suture tape repair shows both durability and limitations. Hopper et al. reported 5-year outcomes after primary repair with suture tape augmentation in 34 patients available for analysis. The mean KOOS improved from 48.7 preoperatively to 88.5 at 5 years, but 6 patients sustained rerupture, corresponding to 17.6%; those who failed were younger and had higher initial Marx activity scores (30). In a larger retrospective comparison of secondary surgery, Hopper et al. compared 134 ACL repair patients with 272 ACL reconstruction patients and found no statistically significant difference in rerupture, secondary meniscal surgery, contralateral ACL rupture, or overall secondary surgery, although rerupture was numerically higher after repair than reconstruction (32).

Other suture-augmentation cohorts report variable outcomes, with recurrent injury and revision rates differing across studies. Douoguih et al. reviewed 27 patients with proximal avulsion or high-grade partial avulsion treated with suture augmentation and reported 4 recurrent ACL injuries requiring revision reconstruction at 2.8-year mean follow-up, while the remaining patients had no clinical instability or subjective complaints (33). Schneider et al. reported 88 patients available for follow-up after primary ACL repair using suture augmentation, with a mean IKDC score of 87.4, mean Lysholm 92.6, and 3% revision surgery at a mean 21 months (34). In a later revision-risk study from the same group, 9 of 86 patients underwent revision surgery, with revision-free survival of 97% at 1 year, 93% at 2 years, and 90% at 4 years (35). Al Kindi et al. reported a prospective selected cohort with minimum 3-year follow-up and an overall success rate of 82.2%; failures were substantially lower in Sherman type I tears than type II tears (31).

Comparative studies suggest similar short-term patient-reported outcomes between repair and reconstruction in selected cohorts, but their interpretation remains methodologically complex. Hoogeslag et al. conducted a randomized trial comparing DIS with single-bundle all-inside ACL reconstruction. The DIS group was noninferior for the IKDC subjective score at 2 years, with median IKDC 95.4 in the repair group vs. 94.3 in the reconstruction group; ipsilateral rerupture occurred in 8.7% vs. 19.0%, respectively, but repeat surgery for other reasons was numerically higher after repair (28). Kösters et al. similarly compared DIS with ACL reconstruction in a randomized study and found comparable functional outcomes at 2 years, although anterior tibial translation was greater after DIS and clinical failure occurred in 16.3% of DIS patients vs. 12.5% of reconstruction patients (15). At 5 years, Glasbrenner et al. reported comparable instrumented laxity and PROMs between DIS and reconstruction, but recurrent instability was observed in 35% of the repair group vs. 20% of the reconstruction group, particularly in younger and high-activity patients (16).

Clinical studies comparing primary ACL repair with static suture tape augmentation against reconstruction have reported similar short-term outcomes in selected repairable tears, although interpretation remains limited by small samples and nonrandomized selection. Szwedowski et al. compared primary repair with internal bracing against anatomic single-bundle reconstruction and found significantly lower anterior tibial translation in the repair group, while Lysholm scores were not significantly different (36). Müller et al. evaluated primary ACL repair using the branded Arthrex InternalBrace™ technique and compared it with matched ACL reconstruction and healthy control groups, reporting comparable patient-reported, clinical, and functional outcomes at 2 years and a shorter operative time in the repair group (37). Duong et al. compared ACL repair with reconstruction using clinical, MRI, and patient-reported outcomes, providing further evidence that selected proximal repairs can achieve outcomes comparable to reconstruction, although full interpretation remains limited by selection and matching issues (38).

Recent comparative work continues to expand the evidence base. Simard et al. compared ACL repair with suture tape augmentation for proximal tears, early ACL reconstruction with suture tape augmentation, and standard ACL reconstruction, reporting comparable short-term clinical outcomes and side-to-side laxity across groups at 2 years (39). Garside et al. evaluated meniscal repair performed with concomitant suture-augmented ACL repair vs. reconstruction and found that meniscal repair with suture-augmented ACL repair was not inferior to meniscal repair with suture-augmented ACL reconstruction at 2 years (27). These studies are useful because they address clinically realistic scenarios, but they still involve selected repairable tears rather than unselected ACL rupture populations (27, 39). The characteristics, principal findings, and methodological limitations of the additional midterm and comparative studies are summarized in Table 3.

Return-to-sport and early recovery data are relevant because one of the proposed advantages of repair is reduced surgical morbidity. Vermeijden et al. reported that among adult patients with preinjury Tegner ≥6, 85% returned to any sport, 70% returned to knee-strenuous sport, and 60% returned to preinjury sport level after arthroscopic primary repair; median return to running was 90 days and median return to sport was 180 days (25). A separate study found that patients had less daily awareness of the operated knee after repair than after reconstruction, as measured by the Forgotten Joint Score-12 (40). In bilateral ACL patients who had reconstruction on one side and repair on the other, many reported less pain, earlier range-of-motion return, and faster rehabilitation progression after repair, despite similar PROMs between knees (41). Prospective pain data also showed that repair patients used fewer opioids than reconstruction patients in the early postoperative period and had better early range of motion and recovery-quality scores (42).

Healing studies provide imaging and arthroscopic support for the biological plausibility of repair. Ferretti et al. prospectively evaluated 10 acute repairs with sequential MRI and found normal ACL morphology in all cases at 1, 3, and 6 months; by 3 and 6 months, 9 of 10 patients had isointense signal and 1 had intermediate signal (22). Heusdens et al. also incorporated MRI healing assessment into a prospective case series and found that grade 3 healing at 6 months was associated with higher rerupture risk (29). Annibaldi et al. used in-office needle arthroscopy after acute ACL repair and found type A healing in 11 patients and type B healing in 4, with good or fair synovial coverage in all cases and significant correlations between arthroscopic healing, ACL tension, MRI appearance, and KT-1000 findings (21).

DIS studies add important preservation evidence but should be interpreted separately from primary repair using static suture tape augmentation. Kayaalp et al. reported that DIS produced clinical outcomes similar to all-inside reconstruction in moderately active patients, with faster psychological recovery reflected by ACL-RSI scores (13). Senftl et al., however, reported a 16.3% reoperation rate for inadequate healing after DIS and an additional group with objective residual laxity, raising concerns about healing reliability (14). Farid et al. reported a 30.2% 1-year graft failure rate using a definition that included rerupture, revision, or >3 mm side-to-side anterior tibial translation, although only 7% required secondary reconstructive surgery (43). Eberlein et al. found a 5-year DIS survival rate of 69.6% and reported that collagen wrapping or local platelet-rich fibrin did not improve survival (44).

Collectively, clinical studies report satisfactory short- to midterm outcomes in selected proximal tears, but interpretation is limited by heterogeneous repair techniques, patient-selection criteria, follow-up durations, and definitions of failure. These limitations prevent firm equivalence conclusions between primary repair and ACL reconstruction.

## 6 Failure predictors and revision concerns

Failure after primary ACL repair is highly dependent on patient selection, tear biology, activity demand, surgical technique, and the definition used for failure. Reported failures may include traumatic rerupture, symptomatic instability, abnormal instrumented laxity, revision ACL reconstruction, or reoperation for implant-related problems. This variability explains why failure rates differ substantially across studies and why direct comparison between cohorts should be cautious.

Young age is one of the most consistent risk signals. Vermeijden et al. specifically examined the role of age after arthroscopic primary repair of proximal ACL tears and found markedly higher failure in patients aged 21 years or younger compared with older patients (23). This is consistent with adolescent-specific evidence. Gagliardi et al. found that ACL repair with suture ligament augmentation in adolescents had a much higher cumulative failure incidence than quadriceps tendon–patellar bone autograft reconstruction, suggesting that biologically repairable morphology alone may not be sufficient in young pivoting athletes (8). Although Bigoni et al. reported no reinjury or growth arrest in five skeletally immature patients at short-term follow-up, that cohort was very small and highly selected, so it should not override the broader caution regarding adolescent patients (12).

High activity demand is another major concern. Cruz et al. reported a 26.1% failure rate in an active-duty military population after ACL repair with suture tape augmentation and identified younger age, elite/competitive or operational activity status, delayed surgery, and active tobacco use as risk factors (24). Hopper et al. also found that rerupture at 5 years after suture tape repair occurred more often in younger patients with higher initial Marx activity scores (30). These findings suggest that repair may be less durable when exposed to early or repeated high-load pivoting activity, even if the tear is technically repairable.

Tear pattern and tissue biology are major determinants of failure. Ateschrang et al. reported failure rates of 4% in one-part tears with intact synovial coverage, compared with 26.9% in two-part tears and 27.3% in multilacerated tears; synovial disruption remained independently associated with failure (19). Al Kindi et al. similarly reported better survival in Sherman type I than type II tears (31). Poor tissue quality, disrupted synovial coverage, multilacerated morphology, insufficient remnant length, or inability to reduce the ligament to the femoral footprint should therefore prompt conversion to ACL reconstruction. Delayed surgery may further increase failure risk by reducing remnant mobility and tissue quality, as discussed in the patient-selection section (5, 22).

Revision concerns differ by repair technique. Failed primary ACL repair with static suture tape augmentation is usually revised to standard ACL reconstruction, but failed DIS may be more complex. Cristiani et al. reported that after failed DIS, only 4 of 10 patients underwent single-stage ACL reconstruction, while 6 required staged management because of arthrofibrosis or tibial tunnel enlargement (45). Senftl et al. also reported a relevant reoperation rate and residual laxity after DIS, while Farid et al. showed that failure rates can be high when objective laxity is included in the definition (14, 43). These findings emphasize that patients should be counseled not only about rerupture risk but also about the potential complexity of revision surgery.

Failure risk appears cumulative rather than attributable to a single factor; age, activity demand, timing of surgery, tear morphology, remnant quality, and synovial integrity should therefore be considered jointly during treatment selection and patient counseling.

## 7 Clinical positioning and practice points

Primary ACL repair with suture tape augmentation should be reserved for patients with a potentially repairable proximal tear on preoperative imaging and satisfactory remnant length, tissue quality, reducibility, and synovial integrity confirmed arthroscopically. ACL reconstruction remains preferable when these criteria are not met or when the patient has a midsubstance or distal tear, a chronic or retracted remnant, poor tissue quality, severe instability, multiligament injury, or very high mechanical demands. The principal selection criteria and findings favoring reconstruction are summarized in Table 1 (4, 5, 17–19).

Patient counseling is essential. Potential advantages of repair include native-ligament preservation, avoidance of graft harvest, less early pain, lower opioid use, earlier range-of-motion recovery, and reduced awareness of the operated knee in some studies (40–42). These potential benefits must be balanced against variable failure rates, particularly in younger and high-demand patients. Patients should also understand that failed repair generally requires ACL reconstruction and that revision after DIS may occasionally require staged surgery (45).

Rehabilitation should remain cautious and criteria-based. Although suture tape augmentation may improve early construct stability, it should not be used to justify accelerated or uncontrolled return to pivoting sport. Return-to-sport decisions should be based on strength symmetry, neuromuscular control, psychological readiness, absence of instability, and satisfactory sport-specific testing. Mechanical augmentation does not eliminate the need to protect biological ligament healing.

## 8 Evidence gaps, future directions, and limitations

The most important evidence gap is the shortage of large randomized trials comparing modern primary ACL repair with static suture tape augmentation against contemporary ACL reconstruction in patients with the same repairable proximal tear patterns. Many existing comparisons are inherently biased because repair is offered primarily to proximal tears with adequate remnant length and tissue quality, whereas reconstruction is used for a broader spectrum of ACL injuries. Therefore, similar outcomes between repair and reconstruction in selected cohorts do not prove that repair is equivalent for all ACL tears (15, 16, 28, 36, 39).

Future trials should standardize indications. At minimum, studies should clearly define Sherman tear type, distal remnant length, tissue quality, synovial coverage, time from injury to surgery, age, sport level, and associated meniscal or anterolateral pathology. Without these details, it is difficult to know whether differences in outcome reflect the repair technique itself or differences in patient selection.

Failure definitions also need standardization. Some studies define failure only as revision ACL reconstruction, while others include rerupture, subjective instability, abnormal Lachman or pivot shift, excessive side-to-side laxity, or MRI non-healing (14, 29, 43). A clinically useful definition should probably separate traumatic rerupture, biological non-healing, symptomatic laxity, objective laxity without symptoms, and reoperation for implant-related reasons. This would make future studies easier to compare.

Long-term durability remains uncertain. Several studies report good 2-year outcomes, but failure may increase over time, especially in young and high-demand patients (16, 30, 46). Future 5–10 year follow-up should assess not only revision rates but also return to preinjury sport, secondary meniscal injury, cartilage degeneration, osteoarthritis, patient satisfaction, and ability to maintain activity.

Prediction models are needed to guide individualized decision-making. Future models should combine age, activity level, BMI, smoking, time to surgery, Sherman type, MRI remnant length, intraoperative synovial coverage, tissue quality, and associated injuries. Imaging and second-look arthroscopy studies may also help determine whether MRI healing grade or synovial coverage predicts long-term survival (22, 29).

Biomechanical research should continue, but laboratory findings must be validated clinically. Current studies show that internal bracing can reduce gap formation, improve load-sharing, and bring knee laxity closer to the intact ACL state (9–11). Other biomechanical work suggests that cortical suspensory fixation may have stronger load-to-failure than knotless anchor repair, and that multiple high-strength sutures may outperform traditional suture tape in time-zero testing (47, 48). However, mechanical strength in a laboratory model does not automatically translate into lower rerupture rates in athletes.

The current evidence base has several limitations. Many studies are Level III or IV, include small cohorts, have short follow-up, and come from expert centers. Techniques vary widely and include suture-anchor repair, static suture tape augmentation, dynamic intraligamentary stabilization, and scaffold-assisted bridge-enhanced ACL restoration. Some cohorts overlap, and several studies use highly selected patients, which limits generalizability. In addition, some newer studies are only available through abstracts or limited online access, restricting detailed evaluation of rehabilitation protocols, complications, and subgroup outcomes (20, 27, 38, 39, 46, 49). In addition, no formal risk-of-bias assessment or certainty-of-evidence grading was performed because of the narrative design and heterogeneity of the included study types. This limits the ability to quantify study-level methodological bias and should be considered when interpreting the strength of the synthesized evidence. Therefore, conclusions should remain cautious and selection-specific.

### 8.1 Related ACL-preservation strategy considered separately: BEAR

Bridge-enhanced anterior cruciate ligament restoration (BEAR), originally described as bridge-enhanced ACL repair, is a biologically assisted preservation strategy that differs from isolated suture-anchor repair, static suture tape augmentation, and dynamic intraligamentary stabilization. The procedure combines suture stabilization of the torn ACL with an extracellular-matrix implant containing autologous blood, which is positioned between the torn ligament ends to facilitate native ligament healing (50).

In a prospective randomized trial of 100 patients with complete midsubstance ACL tears, BEAR was noninferior to autograft ACL reconstruction at 2 years for patient-reported knee function and instrumented anteroposterior laxity. Hamstring strength was greater after BEAR, whereas a second ipsilateral ACL procedure was required in 14% of the BEAR group and 6% of the reconstruction group; this difference was not statistically significant (50).

Longer-term evidence remains limited. In the first-in-human cohort, outcomes were generally similar between BEAR and hamstring-autograft reconstruction at 6 years, apart from greater isometric hamstring strength after BEAR. However, the analysis included only 10 BEAR patients and 10 reconstruction patients at initial enrollment, with additional losses to follow-up (51).

Early postcommercial registry evidence from the first 100 BEAR procedures reported an overall reoperation rate of 8.3% at 1 year and 11.5% at final follow-up, with an ACL retear rate of 2.1% at final follow-up. Mean follow-up was approximately 15 months, so these results provide preliminary safety and short-term outcome data rather than evidence of long-term durability (52).

BEAR therefore represents an important emerging ACL-preservation technique, but its scaffold-assisted biological mechanism and clinical evidence should be evaluated separately from primary ACL repair using isolated suture tape augmentation. Larger independent studies with longer follow-up are required to define its comparative durability, indications, failure predictors, and role relative to ACL reconstruction.

## 9 Conclusion

Primary ACL repair with suture tape augmentation is a selective tissue-preserving option rather than a replacement for reconstruction. The strongest candidates have an acute proximal tear, an adequate reducible remnant, good tissue quality, and preserved synovial coverage. Short- to midterm outcomes can be satisfactory, but failure is more frequent in young and high-demand populations, and long-term comparative evidence remains limited. Treatment should therefore be guided by imaging, arthroscopic confirmation, and transparent patient counseling. Future studies should use standardized indications, technique descriptions, rehabilitation protocols, and failure definitions.
